# *Mastomys natalensis* and Lassa Fever, West Africa

**DOI:** 10.3201/eid1212.060812

**Published:** 2006-12

**Authors:** Emilie Lecompte, Elisabeth Fichet-Calvet, Stéphane Daffis, Kékoura Koulémou, Oumar Sylla, Fodé Kourouma, Amadou Doré, Barré Soropogui, Vladimir Aniskin, Bernard Allali, Stéphane Kouassi Kan, Aude Lalis, Lamine Koivogui, Stephan Günther, Christiane Denys, Jan ter Meulen

**Affiliations:** * Philipps University Institute of Virology, Marburg, Germany;; † Muséum National d'Histoire Naturelle, Paris, France;; ‡ Washington University School of Medicine, Saint Louis, Missouri, USA;; § Projet de Recherches sur les Fièvres Hémorragiques en Guinée, Conakry, Guinea;; ¶ Severtsov Institute of Ecology and Evolution, Moscow, Russia;; # Institut Pasteur d'Abidjan, Abidjan, Côte d'Ivoire;; ** Bernhard Nocht Institute for Tropical Medicine, Hamburg, Germany;; †† Leiden University Medical Center, Leiden, the Netherlands

**Keywords:** Mastomys, Lassa virus, Lassa fever, reservoir host, arenavirus, dispatch

## Abstract

PCR screening of 1,482 murid rodents from 13 genera caught in 18 different localities of Guinea, West Africa, showed Lassa virus infection only in molecularly typed *Mastomys natalensis*. Distribution of this rodent and relative abundance compared with *M. erythroleucus* correlates geographically with Lassa virus seroprevalence in humans.

Arenaviruses are emerging in the Americas and Africa and can cause hemorrhagic fevers with case fatalities of up to 15%. These viruses are mainly transmitted through contact with the excreta of their natural hosts, rodents of the family Muridae. The Old World arenavirus Lassa virus causes up to 300,000 cases of Lassa fever annually in endemic foci of 2 geographically disjunct regions of West Africa (*1*).

Most arenaviruses have been associated with 1 specific reservoir host species (*2*). Knowledge of the geographic distribution of the taxonomically defined host is therefore essential to understand the epidemiology of human infections. In the 1970s, the rodent host of Lassa virus was classified as Mastomys natalensis (*3*); later, when hemoglobin electrophoresis was used for species determination, M. erythroleucus and possibly M. huberti were also proposed as hosts (*1*). In addition, Lassa virus antigen was detected in Rattus and Mus genera, raising the possibility that other rodent genera could be involved in transmission (*4*).

The taxonomy of Mastomys is considered unresolved, and species determination remains problematic; 8 distinct species are recognized, and several coexist in Lassa fever–endemic areas (*5*). The uncertainty about their precise natural host relationships with Lassa virus is considered a major obstacle for a better understanding of the restricted distribution of Lassa fever in West Africa (*6*).

In this study, we molecularly typed >1,000 specimens of Mastomys spp. from Guinea with a recently established species-specific PCR (*7*). The rodents were then screened for Lassa virus infection with a reverse transcription PCR (RT-PCR), which was shown to amplify Lassa virus strains from Sierra Leone, Liberia, Guinea, Côte d'Ivoire, and Nigeria, as well as the African arenaviruses Mobala and Ippy and 3 strains of the related lymphocytic choriomeningitis virus (*8*).

## The Study

In a survey of rodentborne hemorrhagic fever viruses, 1,591 small mammals were trapped in Guinea from 2002 to 2005. Total RNA was extracted from rodent blood preserved in liquid nitrogen by using the Blood RNA kit (Peqlab, Erlangen, Germany). A 1-step RT-PCR targeting a highly conserved region of the RNA polymerase (L) gene was performed by using primers LVL3359A-plus (5´-AGAATTAGTGAAAGGGAGAGCAATTC), LVL3359D-plus (5´-AGAATCAGTGAAAGGGAAAGCAATTC), LVL3359G-plus (5´-AGAATTAGTGAAAGGGAGAGTAACTC), LVL3754A-minus (5´-CACATCATTGGTCCCCATTTACTATGATC), and LVL3754D-minus (5´-CACATCATTGGTCCCCATTTACTGTGATC) (Note: Underlined letters represent differences in nucleotides among plus and minus primers). Because Lassa virus antigen should be frequently detectable in the natural host, and specific antibodies are known to be negatively correlated with its presence, they were not investigated in this study (*9*).

**Figure 1:**
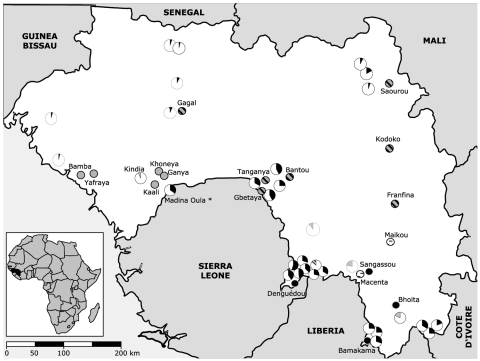
Map of Guinea showing the location of the 18 trapping sites (small circles). Sites where only Mastomys erythroleucus or M. natalensis were trapped are shaded in gray and black, respectively. Sites where both species were captured are hatched, and sites where no Mastomys were caught are marked with a dash. The human Lassa virus seroprevalence in these areas is indicated by the size of the sectors of the larger circles shaded black (*11*), gray (*12*), or hatched (*13*). The asterisk denotes the Madina Oula refugee camp on the border with Sierra Leone, where human seroprevalence was high (*11*), with 23% of the Mastomys Lassa virus antigen or antibody positive (*9*). In close localities, no Lassa virus–positive Mastomys were found (*9*).

The animals were caught in 18 different study sites representative of the principal geographic regions of Guinea (*10*) (Figure 1). All sites were rural villages with a population <1,000. Human Lassa fever seroprevalence was previously reported to be low (0%–11%) in 6 trapping areas and high (25%–55%) in 12 trapping areas (11–13, Figure 1). Of 1,482 murid rodents belonging to 13 genera and at least 20 species, we typed 847 as M. natalensis and 202 as M. erythroleucus but none as M. huberti, by using DNA from liver biopsies. In addition, we karyotyped 12 members of the genus Mastomys in the field by using standard procedures (*14*).

We obtained positive RT-PCR results from 98 (1.2%) of 1,482 murid rodents. Sequence analysis showed 96 Lassa virus strains with 96%–100% amino acid homology with the prototypic strain Josiah. Lassa virus–positive rodents were only captured in the prefectures of Faranah (villages of Gbetaya, Bantou, Tanganya) and Guéckédou (Denguédou), both situated along the border with Sierra Leone (Figure 1). We PCR-typed all Lassa virus–positive rodents unequivocally as M. natalensis, with 1 male (no. BA686) additionally confirmed by karyotyping (2n = 32, autosomal fundamental number = 53). Overall, 11.3% of M. natalensis were infected with Lassa virus (Table), with 0% in the low seroprevalence area and 5.4%–32.1% in the Lassa fever high seroprevalence area. In the coastal region, where the lowest human Lassa virus seroprevalence (0%–6%) has been reported, only M. erythroleucus was captured. In contrast, in the forest region, where the highest seroprevalence of up to 55% in selected villages has been found, only M. natalensis was trapped (*11*). Both species were captured in the savannah regions, where seroprevalence was 2%–42%, but M. natalensis was captured more frequently (Figure 1).

**Table:** Small mammal species examined for arenavirus infection by reverse transcription PCR*

Species	Lassa fever high-prevalence areas		Lassa fever low-prevalence areas		Total	
	No. examined	No. positive (%)	No. examined	No. positive (%)	No. examined	No. positive (%)
Mastomys natalensis	726	96 (13.2)	121	0	847	96 (11.3)
Mastomys erythroleucus	65	0	137	0	202	0
Mus (Nannomys) spp.	131	1 (0.7)	34	1 (5.6)	165	2 (1.2)
Praomys cf. rostratus	78	0	42	0	117	0
Myomys daltoni	24	0	25	0	49	0
Crocidura spp.	22	0	18	0	40	0
Lophuromys sikapusi	18	0	21	0	39	0
Lemniscomys spp.	19	0	10	0	29	0
Rattus rattus	8	0	51	0	59	0
Tatera cf. guinea	3	0	12	0	15	0
Uranomys ruddi	1	0	6	0	7	0
Hylomyscus simus	5	0	0	0	5	0
Malacomys edwardsi	2	0	0	0	2	0
Hybomys spp.	3	0	0	0	3	0
Mus musculus	0	0	1	0	1	0
Cricetomys gambianus	1	0	0	0	1	0
Graphiurus sp.	1	0	0	0	1	0
Paraxerus sp.	1	0	0	0	1	0
Sylvisorex sp.	1	0	0	0	1	0
Micropteropus sp.	0	0	4	0	4	0
Thamnomys sp.	0	0	1	0	1	0
Dasymys rufulus	0	0	1	0	1	0
Lepus sp.	0	0	1	0	1	0
Total/Lassa virus	1,271	96	320	0	1,591	96 (6)
Total/Arenaviruses	1,271	97	320	1	1,591	98 (6.2)

*The Lassa fever high-prevalence areas comprise the region of Faranah and the forest region (Figure 1); the low-prevalence areas comprise all other localities of the study.

**Figure 2:**
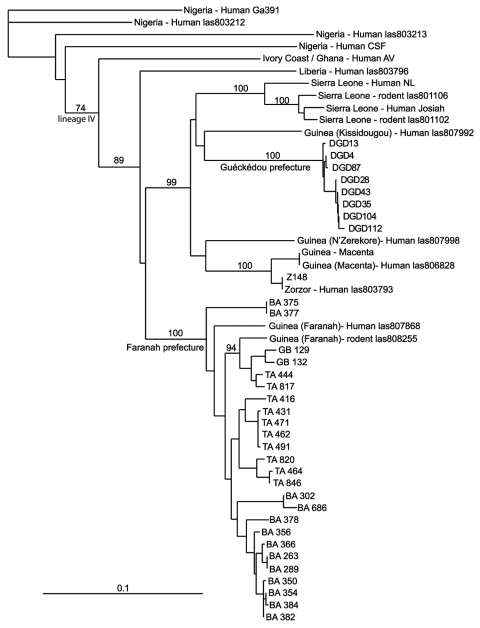
Phylogenetic relationships of Lassa virus strains based on a nucleoprotein gene fragment (631 bp) determined by using the neighbor-joining method. The numbers above branches are bootstrap values >50% (1,000 replicates). Scale bar indicates 10% divergence. Localities are indicated by the specimen label: DGD (Denguédou), BA (Bantou), GB (Gbetaya), and TA (Tanganya).

Lassa virus was isolated in cell culture from 32 rodents, and a 631-bp fragment of the nucleoprotein gene previously used for phylogenetic analysis was sequenced (GenBank accession nos. DQ832667–DQ832699) (*15*). The phylogenetic tree shows that all isolates belong to the lineage IV of Lassa virus and that strains from the prefecture of Faranah cluster with strains isolated previously from human patients of the same region (*15*) (Figure 2). We detected 2 novel L-gene sequences that shared 68%–74% homology with lymphocytic choriomeningitis virus in 2 rodents of the Mus subgenus Nannomys.

## Conclusions

Our results indicate that M. natalensis is very likely the only reservoir host of Lassa virus in Guinea. Previous studies that identified M. erythroleucus and M. huberti as reservoirs of Lassa virus in Sierra Leone may have confused the species, especially because M. natalensis and M. huberti have an identical number of chromosomes (*5**,**7*). Despite a massive trapping effort, M. huberti was not detected in the regions of Guinea bordering Sierra Leone that had high human Lassa virus seroprevalence. Therefore, M. natalensis is likely also the only reservoir in Sierra Leone. Whether this is also the case for the genetically more remote lineage I–III strains of Lassa virus remains to be evaluated because M. kollmannspergeri is also present in Nigeria (*7**,**16*). While it is reassuring that the reservoir for Lassa virus is M. natalensis, as reported in the literature since the 1970s, our study demonstrates that proper taxonomic identification of the host is necessary before drawing inferences about the ecology of Lassa virus infection.

The relative abundance of Mastomys correlated with human Lassa virus seroprevalence in Guinea: M. natalensis was absent from the region with the lowest seroprevalence and was the only Mastomys species caught in the highest seroprevalence region. However, if both M. natalensis and M. erythroleucus were present, human Lassa virus prevalence was either low, intermediate, or high (Figure 1). This is a novel finding, which confirms and expands on 2 previous studies conducted in Guinea that reported a correlation between the absence of Mastomys spp. and a low human Lassa virus seroprevalence (*9**,**12*).

The results of this study have multiple implications for explaining the patchy occurrence of Lassa virus in Guinea and neighboring countries, as well as for Lassa fever control. First, assuming that M. natalensis is the only host of Lassa virus, natural nidality may occur in a similar fashion as that described for Bolivian hemorrhagic fever caused by Machupo virus. The Machupo virus reservoir host, Callomys callosus, has been shown by genetic analysis to be paraphyletic. The populations of rodents responsible for the maintenance and transmission of Machupo virus were monophyletic lineages different from C. callosus in non-disease–endemic regions of Bolivian hemorrhagic fever and coevolving with the virus (*17*). M. natalensis specimens from regions with high and low Lassa virus activity are being genetically investigated to determine lineages and population structure. Second, the geographic region of potential rodent-to-human transmission of lineage IV strains of Lassa virus is most likely defined by the occurrence of M. natalensis. Mastomys collections from Sierra Leone and Liberia could be molecularly retyped to reevaluate areas of potential Lassa fever reemergence. Third, our mass screening for arenaviruses found only Lassa virus in Mastomys and a novel lymphocytic choriomeningitislike virus in Mus spp. of the subgenus Nannomys, which are closely related to Mus musculus, the Eurasian host of lymphocytic choriomeningitis virus. These findings support the hypothesis of a species-specific association of arenaviruses with their rodent hosts, resulting in cophylogeny.
